# Identifying associations between sedentary time and cardio-metabolic risk factors in working adults using objective and subjective measures: a cross-sectional analysis

**DOI:** 10.1186/1471-2458-14-1307

**Published:** 2014-12-19

**Authors:** Takanori Honda, Sanmei Chen, Hiro Kishimoto, Kenji Narazaki, Shuzo Kumagai

**Affiliations:** Department of Behavior and Health Sciences, Graduate School of Human-Environment Studies, Kyushu University, 6-1 Kasuga kouen, Kasuga City, Fukuoka Prefecture, 816-8580 Japan; Department of Environmental Medicine, Graduate School of Medical Science, Kyushu University, Maidashi 3-1-1, Higashi-ku, Fukuoka City, Fukuoka Prefecture, 812-8582 Japan; Faculty of Arts and Science, Kyushu University, 6-1 Kasuga kouen, Kasuga City, Fukuoka Prefecture, 816-8580 Japan

**Keywords:** Sedentary behavior, Physical activity, Accelerometry, Self-report questionnaire, Cardiovascular risk factors, Workers

## Abstract

**Background:**

Sedentary behavior has been reported to be associated with metabolic and vascular health independent of moderate-to-vigorous physical activity (MVPA). In order to select appropriate options to measure sedentary behavior in practice and research settings, it is worthwhile to characterize the extent to which objective and subjective measures of sedentary behavior quantify adverse health risks in the same population. This cross-sectional analysis compared accelerometer-derived and self-reported sedentary time to identify their association with cardio-metabolic risk factors.

**Methods:**

Cross-sectional analysis was conducted using data from 661 Japanese workers (145 women) aged 20–64 years. Participants wore a tri-axial accelerometer device for 10 consecutive days and completed the Japan Atherosclerosis Longitudinal Study Physical Activity Questionnaire. Data on body mass index, waist circumference, resting blood pressure, triglycerides, high-density lipoprotein (HDL) and low-density lipoprotein (LDL) cholesterol, total:HDL cholesterol ratio, blood glucose, and glycosylated hemoglobin (HbA1c) were obtained from annual health examinations.

**Results:**

Both accelerometer-derived and self-reported sedentary time were deleteriously associated with triglycerides, HDL-cholesterol, total:HDL ratio, and HbA1c after adjustment for potential confounders including MVPA. There were no significant differences in regression coefficients between the two measures. Thus, the magnitude of the associations of both measures with cardio-metabolic risk factors was similar, despite poor agreement between them. Occupational sedentary time was correlated with both measures of total sedentary time, and more consistently associated with cardio-metabolic risk factors than sedentary leisure time.

**Conclusions:**

Both accelerometer and self-report measurements are similarly associated with cardio-metabolic risk factors in a Japanese working adult population. Subjective and objective measures of sedentary behaviors appear to capture different aspects of behaviors. Further efforts to establish data processing methods integrating objective and subjective measures are needed to more effectively assess sedentary time’s relationship to health outcomes.

**Electronic supplementary material:**

The online version of this article (doi:10.1186/1471-2458-14-1307) contains supplementary material, which is available to authorized users.

## Background

Sedentary behavior, characterized by prolonged periods of activity involving sitting or reclining, has been reported to be associated with metabolic and vascular health outcomes independent of moderate-to-vigorous physical activity (MVPA) [1]. Recent studies showed greater time spent in sedentary behaviors linked to increased risk of mortality from all causes and cardiovascular diseases [2], diabetes [3], metabolic syndrome [4], and impaired glucose and lipid metabolism [5, 6].

Self-report questionnaires are the most commonly used tools to assess sedentary behaviors due to their feasibility in large-scale studies and coverage of information on behavioral contexts [7, 8]. However, self-report measures are prone to recall error and over-reporting, which may distort associations of behavior with outcomes and can lead to erroneous results. More recently, device-based measures, particularly accelerometry, are becoming more commonly used in epidemiological studies as they are highly reliable [8, 9]. While accelerometers are capable of assessing the intensity of human body movements in activity, they often do not take activity posture into account [10].

In order to select appropriate options to measure sedentary behavior in practice and research settings, ability to identify associations with health outcomes is one of the critical criteria that should be considered. From this standpoint, it is worthwhile to characterize the extent to which objective and subjective measures of sedentary behaviors quantify adverse health risks in the same population. Although some previous studies have examined associations of accelerometer-derived and self-report measured sedentary time with cardio-metabolic risk factors in the same adult population, the results were inconsistent [11, 12]. A study from the United Kingdom reported that self-reported sitting time was associated with a number of metabolic risk factors, whereas objectively measured sedentary behaviors were associated with only total cholesterol [11]. By contrast, a study of Chilean adults using accelerometry and the International Physical Activity Questionnaire (IPAQ) reported that both objective and self-reported measures of sedentary time were associated with various risk factors for metabolic and vascular disease [12]. The inconsistency in the findings of these previous studies suggests that a need remains for further comparisons of the associations of different measures of sedentary behavior with cardio-metabolic outcomes.

In a sample of Japanese working adults, we examined the extent to which accelerometer-derived and self-reported sedentary time were each associated with cardio-metabolic risk factors. Prolonged sitting at work is now recognized as an occupational health risk for sedentary office workers. Furthermore, note that the link between sedentary behavior and subsequent health outcomes seems to differ across countries. Given that the prevalence of obesity in Japan is very low despite the high self-reported sitting time [13], data from this population may provide unique information in this context. However, to our knowledge, data regarding sedentary time and cardio-metabolic risk factors in the Japanese population are still limited. The primary purpose of this cross-sectional analysis was to compare associations of sedentary time derived from accelerometer and self-report measurements with cardio-metabolic risk factors in a Japanese working adult population. To further compare these two measurements directly, we examined the extent to which they correlated and agreed with each other in the same population.

## Methods

### Design

This cross-sectional study was conducted among office workers from two Japanese enterprise groups consisting of 12 companies in Japan. The main businesses of the enterprise groups were laboratory testing and food and environmental inspection and information technology. The data were collected between January and March 2010, when an annual health examination was conducted at each enterprise in accordance with the Industrial Safety and Health Act. This study was in accordance with the Declaration of Helsinki, and approved by the ethics committee of the Institute of Health Science, Kyushu University, Fukuoka, Japan. All participants provided written informed consent.

### Participants

A total of 823 full-time workers aged 20 to 64 years from the two enterprise groups were included as the target population in the present study. Of the initial sample, 13 individuals with missing data on self-reported sitting time, 14 individuals without valid accelerometer data, and 7 with missing values for biomarkers were excluded. Individuals with triglycerides ≥400 mg/dL were further excluded due to a known limitation of Friedewald’s estimation as described below (n = 6). In addition, 77 individuals with missing data on other covariates were excluded. Finally, 661 participants (145 women) were included in the analyses.

### Objective measurement of sedentary time

Daily sedentary time was assessed using a tri-axial accelerometer device (Active Style Pro HJA 350-IT, Omron Healthcare Co. Ltd., Kyoto). Since participants may have voluntarily modified their lifestyle behaviors after participating in the health examination, we sent accelerometers to participants prior to the health examination. Participants were given instructions to wear the device during waking hours for 10 consecutive days, except while bathing or sleeping. Data were recorded in 60-second epochs. In this study, we defined objective sedentary behavior according to activity intensity (≤1.5 metabolic equivalents [METs]) [14] and calculated daily sedentary time as minutes/day. Intensity of minute-by-minute activity was estimated by built-in algorithms containing a specific equation for sedentary activities [15]. The accuracy of the intensity estimation has been validated with the Douglas bag method [15, 16]. Non-wear time was defined as time periods of at least 60 consecutive minutes of no activity (i.e., estimated activity intensity < 1.0 MET) with allowance for up to two consecutive minutes of activities with intensity equal to 1.0 MET. With respect to intensity threshold of non-wear time, previous studies employed the same threshold with sedentary behavior (i.e., typically 100 counts per minute or 1.5 METs). Since the tri-axial accelerometer we used is highly sensitive and valid for estimating low-intensity activities, it enabled us to employ 1.0 MET as the threshold for non-wear-time. We used the SAS macro program provided by the National Institute of Cancer to compute daily wearing time, with modifications based on our accelerometer [17]. Days with at least 600 minutes of wearing time were considered valid [18]. Participants had to have at least four valid days to be included in the analyses [7].

### Subjective measurement of sedentary time

Subjective sedentary time was assessed with the Japan Arteriosclerosis Longitudinal Study Physical Activity Questionnaire (JALSPAQ). This measure was developed to assess daily amount of physical activity for the general Japanese population and has been validated against total energy expenditure, using the doubly labeled water technique [19]. The JALSPAQ consists of 14 detailed questions in five domains (sleep, occupational activity, transportation, housework, and leisure time activity) [19]. Two domains (occupational and leisure time activity) include questions about sedentary behaviors. Overall sedentary time was calculated as the sum of leisure time and occupational sedentary time. Time spent in sedentary activity during leisure time was assessed by a single item from the JALSPAQ: “How many hours do you usually stay sedentary every day, such as television viewing, reading, listening to music, playing board games, and using the computer, in leisure time?” Similar questions on leisure time sitting have been used in other population studies [20, 21]. Occupational sedentary time was estimated based on the standard procedure for the JALSPAQ [19], from responses to two questions regarding working hours and proportion of time spent in sitting during work. Information on working hours was obtained through the following question asking: “How many hours per week do you usually work?” Proportion of time spent in sitting during work was rated as follows: 1) almost all of the time, 2) more than half of working hours, 3) approximately half of working hours, 4) less than half of working hours, or 5) almost none of the time. A proportion coefficient was given corresponding to the chosen answer as 1.0, 0.75, 0.5, 0.25, and 0, respectively. Participants were also asked to report their working hours. Occupational sedentary time was then calculated by working hours multiplied by the given proportion coefficient. Since this estimation method of occupational sedentary time has not been validated against a criterion measurement, we also coded the original Likert scale as a continuous variable in the analyses.

### Cardio-metabolic risk factors

All cardio-metabolic risk factor data were obtained from the annual health examinations. Specialized health-examination nurses measured height, weight, waist circumference, and blood pressure using standard protocols. Body mass index (BMI) was calculated as weight divided by height squared (kg/m^2^). Waist circumference was measured to the nearest 0.1 cm. Systolic and diastolic blood pressure was measured at rest by an automated sphygmomanometer. Blood samples were analyzed for triglycerides, total and high-density lipoprotein (HDL) cholesterol, and blood glucose by enzymatic methods and glycosylated hemoglobin (HbA1c) by latex agglutination methods or high performance liquid chromatography. Total:HDL cholesterol ratio was subsequently calculated. Low density lipoprotein (LDL) cholesterol was calculated using the Friedewald formula [22]. All participants were asked to fast overnight before the blood test.

### Covariates

Information about educational attainment (more or less than college education), current smoking and drinking habits (yes/no), and marital status (married, not married) were obtained from a self-report questionnaire. Occupation was classified into the following categories: managers, professionals, clerks, sales, and others. Total calorie intake and saturated fat consumption in the past month were assessed using the brief-type self-administered diet history questionnaire, which was validated and commonly used in the Japanese adult population to assess dietary habits and nutrition intake [23, 24]. Use of antihypertensive, anti-diabetic, and lipid lowering medications were confirmed by physicians in the health examination. Depressive symptoms were measured by the Center for Epidemiological Studies Depression Scale (CES-D) [25]. Volume of MVPA was measured using the accelerometer and defined as activity intensity ≥ 3 METs. Daily amount of MVPA was calculated as MET multiplied by number of hours in a day. Self-reported MVPA was also assessed and quantified as MET · hours/day using a set of items from the JALSPAQ, which included type, frequency (number of days in a month), and duration (minutes/day) of habitual exercise and other leisure time activities. A MET value was assigned to each self-reported activity according to the 2011 Compendium of Physical Activities by Ainsworth and colleagues [26]. We did not include MVPA at work because it is highly correlated with sedentary time at work, and thus it is possible to over-adjust as previously suggested [27]. We conducted sensitivity analyses where models were adjusted for MVPA during leisure time plus other domains (transportation and housework), and results changed little (data not shown).

### Statistical analyses

All statistical analyses were performed with the SAS software version 9.3 (SAS Institute, Cary, NC, USA) with a significance level of α = 0.05. Data were expressed as mean ± standard deviation (SD), or frequency and percentage. To show participant characteristics by levels of sedentary time, descriptive statistics were computed by tertiles of both accelerometer-derived and self-reported sedentary time, where the cut-off values were 7.99 and 9.76 hours/day for accelerometer and 7.14 and 9.28 hours/day for self-report, respectively.

To assess associations of subjective (self-report) and objective (accelerometer) indicators of sedentary time with cardio-metabolic risk factors, multiple linear regression analyses were performed. Triglycerides, blood glucose, and HbA1c were logarithmically transformed in order to normalize their skewed distributions. The first model adjusted for sex and age. In the second model, we additionally adjusted for educational attainment, marital status, smoking and drinking status, total calorie intake, saturated fat consumption, use of medication, and occupation. The third model further adjusted for accelerometer-assessed MVPA or self-reported MVPA, respectively, to examine whether the associations were independent of MVPA. Accelerometer wearing time was also adjusted in models where accelerometer-derived sedentary time was included as an independent variable. Further, we repeated multiple linear regression analyses with specific domains of self-reported sedentary time (leisure time and occupational) entered as independent variables. Multicollinearity for variables in each model was tested using the variance inflation factor (VIF) test. In our models, acceptable VIF value was no greater than 3 for all covariates. For the markers which were associated with both measures, we further conducted the Wald test to compare regression coefficients of accelerometer-derived and total self-reported sedentary time to determine whether magnitude of associations differed. The null hypothesis was that there was no difference between those two standardized regression coefficients. Furthermore, an interaction term was fitted to assess whether the effects of accelerometer-derived sedentary time on cardio-metabolic factors varied across self-perceived levels of sedentary behavior, and vice versa. Both independent variables were mean centered and an interaction term between two measures was included in the same model. Agreement between accelerometer-derived and self-reported sedentary time was examined using the method outlined by Bland and Altman [28]. Systematic and random error between two measurements was assessed using a linear regression model. Spearman’s correlation coefficient rho was calculated for each variable of sedentary time.

## Results

### Characteristics of the study participants

Additional file 1: Table S1 presents the characteristics of the participants by tertiles of sedentary time derived from the accelerometer and the self-report questionnaire (see Additional file 1: Table S1). The mean age of the participants was 43 ± 9 years and 78% were men. The participants reported sedentary time of 8.4 ± 3.4 hours/day on average in the questionnaire, and the mean sedentary time recorded by the accelerometer was 8.8 ± 2.2 hours/day with wearing time of 14.2 ± 1.6 hours/day. There was an increasing trend of objective sedentary time with older age, higher proportion of men, being married, higher education level, increased level of total and occupational sedentary time and MVPA measured by self-report, and decreased levels of MVPA measured by accelerometry. Total subjective sedentary time showed a significant trend with greater saturated fat consumption, longer leisure time and occupational sedentary time, and less objective MVPA.

### Association of accelerometer-derived and self-reported sedentary time with cardio-metabolic risk factors

Additional file 1: Table S2 presents the distribution of the cardio-metabolic risk factors according to the levels of sedentary time derived from two measurements (see Additional file 1: Table S2). Both self-reported and accelerometer derived sedentary time showed a significant trend with increased levels of triglycerides, blood glucose, higher total-HDL ratio, and decreased levels of HDL-cholesterol. Additionally, accelerometer-determined sedentary time showed a significant trend with BMI and HbA1c.

As shown in Tables 1 and 2, both self-reported and accelerometer-derived sedentary time showed detrimental associations with triglycerides, HDL-cholesterol, total:HDL ratio, and HbA1c, after adjusting for potential confounders including MVPA (Model 3). Although the regression coefficients for accelerometry were greater than those of self-report, the Wald test showed no difference between the coefficients except for regression on triglycerides (p = 0.012), and no significant interactions were found between two measures across the outcomes. In addition, significant associations were observed between objective sedentary time and BMI and between subjective sedentary time and blood glucose.

**Table 1:** **Multivariate linear regression analysis for accelerometer-derived sedentary time and cardio-metabolic risk factors**

	Model 1			Model 2			Model 3		
	Coefficient	95% CI	P	Coefficient	95% CI	P	Coefficient	95% CI	P
Body mass index	0.119	(-0.023 to 0.261)	0.101	0.118	(-0.032 to 0.267)	0.124	0.213	(0.039 to 0.387)	0.017
Waist circumference	0.282	(-0.132 to 0.696)	0.181	0.262	(-0.172 to 0.696)	0.234	0.237	(-0.257 to 0.731)	0.347
Systolic blood pressure	-0.356	(-0.944 to 0.231)	0.234	-0.188	(-0.794 to 0.418)	0.542	-0.321	(-1.027 to 0.385)	0.372
Diastolic blood pressure	-0.206	(-0.671 to 0.259)	0.385	-0.194	(-0.678 to 0.291)	0.433	-0.119	(-0.684 to 0.446)	0.679
Triglycerides	0.048	(0.027 to 0.068)	<0.001	0.055	(0.033 to 0.076)	<0.001	0.046	(0.021 to 0.071)	<0.001
HDL-Cholesterol	-1.200	(-1.824 to -0.576)	<0.001	-1.381	(-2.045 to -0.717)	<0.001	-1.312	(-2.086 to -0.537)	<0.001
Total:HDL ratio	0.061	(0.021 to 0.101)	0.003	0.085	(0.043 to 0.127)	<0.001	0.072	(0.023 to 0.121)	0.004
LDL-Cholesterol	-0.350	(-1.602 to 0.902)	0.583	0.595	(-0.734 to 1.924)	0.380	0.576	(-0.971 to 2.123)	0.465
Blood glucose	0.002	(-0.003 to 0.008)	0.421	0.005	(-0.0001 to 0.011)	0.063	0.006	(-0.001 to 0.012)	0.088
HbA1c	0.008	(0.002 to 0.014)	0.010	0.008	(0.003 to 0.013)	0.004	0.009	(0.003 to 0.015)	0.006

*Abbreviations*: *CI* confidence interval, *HDL-cholesterol* high-density lipoprotein cholesterol, *LDL-cholesterol* low-density lipoprotein cholesterol, *HbA1c* glycosylated hemoglobin.

Regression coefficients beta corresponding to the mean difference per 60 minutes/day greater sedentary time. Model 1 was adjusted for sex, age, and time accelerometer worn. Model 2 was adjusted for above covariates and educational attainment, marital status, smoking and drinking status, total calorie intake, saturated fat consumption, use of medication, and occupation. Model 3 was adjusted for the same covariates as Model 2 and moderate-to-vigorous physical activity (MVPA). N = 579 for waist circumference, N = 354 for HbA1c, and N = 655–661 for other outcomes due to missing values. Data on triglyceride, blood glucose, and HbA1c were log-transformed.

**Table 2:** **Multivariate linear regression analysis for self-reported sedentary time and cardio-metabolic risk factors**

	Model 1			Model 2			Model 3		
	Coefficient	95% CI	P	Coefficient	95% CI	P	Coefficient	95% CI	P
Body mass index	0.026	(-0.048 to 0.100)	0.492	0.007	(-0.067 to 0.082)	0.850	0.007	(-0.067 to 0.082)	0.849
Waist circumference	0.050	(-0.157 to 0.257)	0.635	-0.016	(-0.226 to 0.194)	0.882	-0.016	(-0.226 to 0.194)	0.878
Systolic blood pressure	-0.081	(-0.387 to 0.224)	0.602	-0.097	(-0.397 to 0.204)	0.528	-0.104	(-0.404 to 0.196)	0.495
Diastolic blood pressure	0.077	(-0.167 to 0.320)	0.537	0.061	(-0.180 to 0.302)	0.621	0.059	(-0.182 to 0.300)	0.632
Triglycerides	0.013	(0.003 to 0.024)	0.015	0.012	(0.001 to 0.022)	0.035	0.012	(0.001 to 0.022)	0.037
HDL-Cholesterol	-0.406	(-0.734 to -0.079)	0.015	-0.437	(-0.770 to -0.105)	0.010	-0.434	(-0.767 to -0.102)	0.011
Total:HDL ratio	0.021	(-0.0002 to 0.041)	0.052	0.025	(0.004 to 0.046)	0.018	0.025	(0.004 to 0.046)	0.020
LDL-Cholesterol	-0.078	(-0.721 to 0.565)	0.812	0.159	(-0.496 to 0.813)	0.634	0.159	(-0. 496 to 0.814)	0.634
Blood glucose	0.004	(0.001 to 0.007)	0.013	0.004	(0.001 to 0.007)	0.003	0.004	(0.001 to 0.007)	0.004
HbA1c	0.004	(0.001 to 0.007)	0.016	0.003	(0.0001 to 0.006)	0.040	0.003	(0.0001 to 0.006)	0.042

*Abbreviations*: *CI* confidence interval, *HDL-cholesterol* high-density lipoprotein cholesterol, *LDL-cholesterol* low-density lipoprotein cholesterol, *HbA1c* glycosylated hemoglobin.

Regression coefficients beta corresponding to the mean difference per 60 minutes/day greater sedentary time. Model 1 was adjusted for sex and age. Model 2 was adjusted for above covariates and educational attainment, marital status, smoking and drinking status, total calorie intake, saturated fat consumption, use of medication, and occupation. Model 3 was adjusted for the same covariates as Model 2 and moderate-to-vigorous physical activity (MVPA). N = 577 for waist circumference, N = 350 for HbA1c, and N = 653–659 for other outcomes due to missing values. Data on triglyceride, blood glucose, and HbA1c were log-transformed.

Table 3 shows different associations of sedentary time during occupation and leisure time with risk factors. Sedentary time during leisure time was only associated with total:HDL cholesterol ratio, while occupational sedentary time was associated with increasing levels of triglycerides, glucose, and HbA1c, and decreasing levels of HDL-cholesterol, after adjustment for all covariates. Similar results were found when occupational sedentary time was included in the model as a continuous variable (results not presented). Mutual adjustment for leisure time and occupational sedentary behavior did not alter the significance of the associations.

**Table 3:** **Multivariate linear regression analysis for leisure time and occupational sedentary behavior and cardio-metabolic risk factors**

	Model 1			Model 2			Model 3		
	Coefficient	95% CI	P	Coefficient	95% CI	P	Coefficient	95% CI	P
Leisure time									
Body mass index	0.032	(-0.066 to 0.129)	0.525	0.009	(-0.089 to 0.107)	0.857	0.009	(-0.089 to 0.107)	0.854
Waist circumference	0.134	(-0.138 to 0.407)	0.334	0.060	(-0.212 to 0.333)	0.664	0.059	(-0.214 to 0.332)	0.670
Systolic blood pressure	0.242	(-0.163 to 0.647)	0.241	0.118	(-0.277 to 0.514)	0.557	0.100	(-0.295 to 0.495)	0.621
Diastolic blood pressure	0.256	(-0.066 to 0.577)	0.119	0.181	(-0.136 to 0.498)	0.263	0.176	(-0.141 to 0.494)	0.276
Triglycerides	0.010	(-0.004 to 0.024)	0.179	0.007	(-0.007 to 0.022)	0.320	0.007	(-0.007 to 0.021)	0.335
HDL-Cholesterol	-0.135	(-0.571 to 0.300)	0.542	-0.175	(-0.614 to 0.264)	0.433	-0.167	(-0.607 to 0.272)	0.455
Total:HDL ratio	0.028	(0.0004 to 0.055)	0.047	0.029	(0.001 to 0.056)	0.041	0.028	(0.001 to 0.056)	0.046
LDL-Cholesterol	0.753	(-0.968 to 1.602)	0.082	0.767	(-0.090 to 1.624)	0.079	0.769	(-0.089 to 1.628)	0.079
Blood Glucose	0.004	(0.0003 to 0.008)	0.035	0.003	(0.0001 to 0.007)	0.055	0.003	(0.0003 to 0.007)	0.071
HbA1c	0.002	(-0.002 to 0.007)	0.355	0.00003	(-0.004 to 0.004)	0.947	0.0001	(-0.004 to 0.004)	0.960
Occupational									
Body mass index	0.019	(-0.096 to 0.133)	0.747	0.005	(-0.113 to 0.123)	0.935	0.005	(-0.113 to 0.123)	0.936
Waist circumference	-0.066	(-0.388 to 0.256)	0.687	-0.134	(-0.471 to 0.204)	0.437	-0.133	(-0.471 to 0.205)	0.439
Systolic blood pressure	-0.525	(-0.997 to -0.053)	0.029	-0.415	(-0.891 to 0.061)	0.087	-0.407	(-0.882 to 0.068)	0.093
Diastolic blood pressure	-0.165	(-0.541 to 0.211)	0.389	-0.110	(-0.492 to 0.273)	0.574	-0.108	(-0.490 to 0.275)	0.580
Triglycerides	0.019	(0.002 to 0.035)	0.027	0.019	(0.002 to 0.036)	0.032	0.019	(0.002 to 0.036)	0.031
HDL-Cholesterol	-0.789	(-1.294 to -0.283)	0.002	-0.845	(-1.371 to -0.320)	0.002	-0.849	(-1.375 to -0.324)	0.002
Total:HDL ratio	0.011	(-0.021 to 0.044)	0.493	0.021	(-0.012 to 0.056)	0.198	0.022	(-0.011 to 0.056)	0.192
LDL-Cholesterol	-1.231	(-2.228 to -0.224)	0.016	-0.734	(-1.780 to 0.313)	0.169	-0.734	(-1.780 to 0.313)	0.169
Blood glucose	0.003	(-0.001 to 0.008)	0.164	0.005	(0.001 to 0.010)	0.017	0.005	(0.001 to 0.010)	0.015
HbA1c	0.007	(0.002 to 0.012)	0.007	0.008	(0.003 to 0.012)	0.001	0.008	(0.003 to 0.012)	0.001

*Abbreviations*: *CI* confidence interval, *HDL-cholesterol* high-density lipoprotein cholesterol, *LDL-cholesterol* low-density lipoprotein cholesterol, *HbA1c* glycosylated hemoglobin.

Regression coefficients beta corresponding to the mean difference per 60 minutes/day greater sedentary time. Model 1 was adjusted for sex and age. Model 2 was adjusted for above covariates and educational attainment, marital status, smoking and drinking status, total calorie intake, saturated fat consumption, use of medication, and occupation. Model 3 was adjusted for the same covariates as Model 2 and moderate-to-vigorous physical activity (MVPA). N = 577 for waist circumference, N = 350 for HbA1c, and N = 653–659 for other outcomes due to missing values. Data on triglyceride, blood glucose, and HbA1c were log-transformed.

### Agreement of accelerometer-derived and self-reported sedentary time

**Figure 1:**
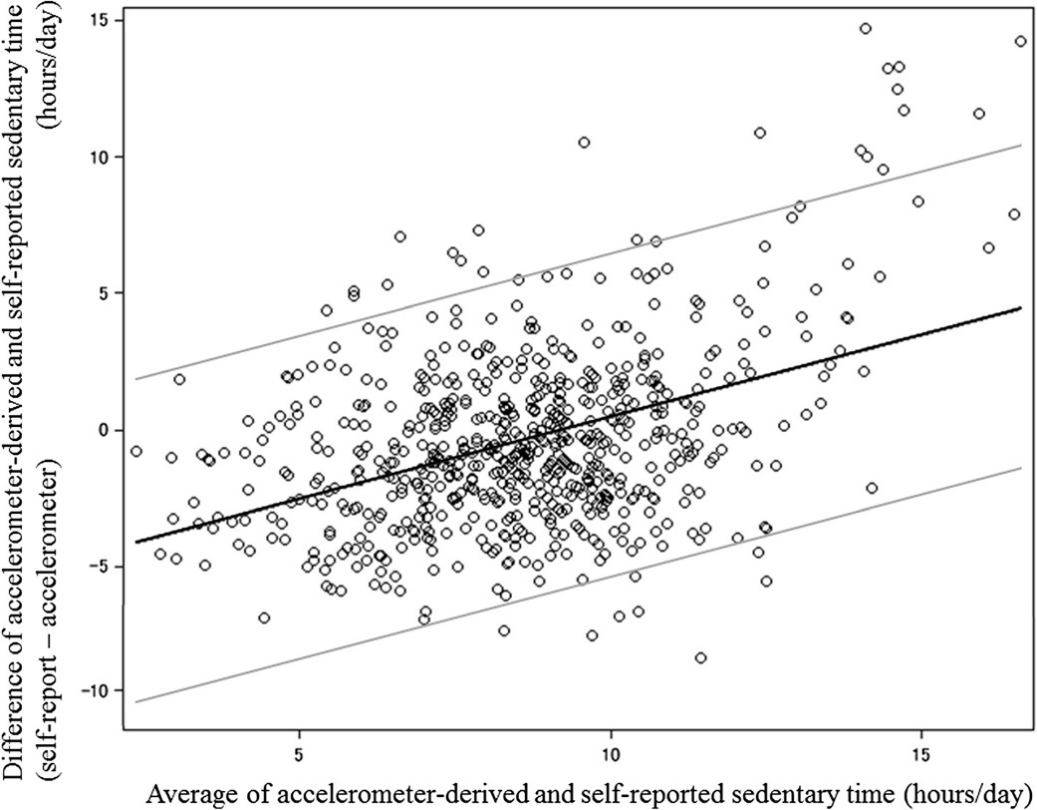
**Bland-Altman plot for accelerometer-derived and self-reported sedentary time.** Solid black line indicates regression line for difference and average of two measures. Gray lines indicate the 95% limits of agreement.

As shown in the Bland-Altman plot (Figure 1), the 95% limits of agreement ranges were wide (mean difference ± 6.3 hours). In addition, the difference in the two indicators increased as the average of two measures increased (beta = 0.602, p < 0.001), indicating that there was proportional bias between two measures. The correlation between accelerometer-determined and self-reported sedentary time was moderate (Spearman’s rho = 0.401, p < 0.001). In the domain-specific analysis, accelerometer-derived sedentary time was significantly correlated with occupational sedentary time (rho = 0.486, p < 0.001) but not with leisure sedentary time (rho = 0.036, p = 0.351), while self-reported total sedentary time was significantly correlated with sedentary time in both domains (occupational, rho = 0.711, p < 0.001; leisure, rho = 0.601, p < 0.001).

## Discussion

In the present study, we investigated the associations of sedentary time derived from accelerometer and self-report questionnaires with cardio-metabolic risk factors and compared the two measurements to identify the association in a working adult population. The main finding of our study was that both tri-axial accelerometer-derived and self-reported sedentary time were deleteriously associated with cardio-metabolic risk factors. Importantly, sedentary time derived from the two measurements was associated with the outcomes in similar magnitude despite the poor agreement between the two measures.

Previous studies comparing associations of different measures of sedentary time with cardio-metabolic risk factors were conducted in the United Kingdom [11] and Chile [12]. The present study extended the previous research by using data from an Asian country. In addition, the results of this study were consistent with a number of population-based studies using self-report measures [29, 30] or uni-axial accelerometer devices [31–33]. We further extended the previous literature by using a tri-axial accelerometer device. Nevertheless, the accelerometer device we used has not been commonly used in previous literature. Thus, the results of this study cannot be directly compared to other studies using different devices. Our study and previous research [6, 30, 32–34] showed similar associations of the current two indicators of sedentary time with lipid and glucose levels. This suggests that the associations partly reflect underlying physiological processes linked to excessive sitting. Prolonged periods of sedentary behavior has been linked to suppression of lipoprotein lipase (LPL) activity in skeletal muscle due to loss of local muscle contraction, which may be the possible pathway underlying the relationship of sedentary time and vascular and metabolic diseases [35]. Previous experimental research reported that low LPL activity was associated with reduced triglyceride uptake and decreased HDL-cholesterol levels [36]. Our epidemiological findings partly support these results.

Although we observed a moderate correlation between accelerometer-derived and self-reported sedentary time, the agreement between the two measures was poor. In this study, since self-reported sedentary time consisted of occupational and leisure time, this poor agreement may have been due to lack of information on transportation or other domains of sedentary behaviors. Despite this, magnitude of the associations of sedentary time did not generally differ between the accelerometer and questionnaire. This may be due to the difference in conceptual focus between the two measures. Contrary to the recent definition, which defines sedentary behavior in terms of both intensity and posture [14], an accelerometer and a questionnaire assess only one of these elements. We found no interactions between the two measures. This indicates that the associations of the two measures with outcomes were not moderated by each other; consequently, they might capture different aspects of sedentary behaviors and be independently associated with cardio-metabolic risk factors. Additionally, as a previous report using past-day recall suggested, short bouts of sitting are unlikely to be recalled [37]. Self-report measurement may be prone to capture prolonged periods of posture in sitting/lying down and ignore short intermittent periods. Thus, it may reflect habitual patterns of sedentary behavior rather than actual time. Proportional error between the two measurements might be explained by this assumption. In other words, although both measures were associated with cardio-metabolic risk factors, sedentary time assessed by these two methods might not be conceptually the same. Therefore, future studies are needed to develop standards of integrating the two methods to operationally define sedentary time.

Our data showed that sedentary time in occupation was consistently correlated with both accelerometer-derived and overall self-reported sedentary time, suggesting that sitting at a workplace may be a main contributor to daily sedentary time among workers. We also observed that sedentary time in occupation was more consistently associated with cardio-metabolic risk factors than that in leisure time. Prior reports suggested that office workers spend their working time in sedentary activity in a more sustained manner with infrequent breaks than in non-occupational time [38]. In light of the increased detrimental effect of longer bouts of sitting on adverse health outcomes [39], workers in the present study might tend to sit in a prolonged manner during working time and thus presented greater associations in occupational sedentary time with the outcomes. Because of the lack of behavioral logs during accelerometer assessment, we could not compare actual bouts of sedentary time during occupation and leisure time.

There are several strengths in our study. First, we used a tri-axial accelerometer to assess sedentary behavior, which is able to estimate physical activity intensity more accurately by its specific algorithm for low-intensity physical activities compared to conventional uni-axial accelerometers [40, 41]. Second, we were able to adjust for a variety of socioeconomic and lifestyle confounders, including occupation, depression, and diet, which were not always available in other population-based studies.

Our study also has several limitations. Its cross-sectional nature precludes examination of causal relationships between sedentary behaviors and outcomes; thus, our results should be interpreted with caution. Since the participants were enrolled from only two enterprise groups, the generalizability of this study is limited. In addition, we did not differentiate between workday and non-workday when quantifying the levels of sedentary time. With respect to measurements, device-based measurement may include some modest misclassification of sitting and standing behavior, and some moderate-to-vigorous intensity physical activities, such as swimming and riding, were not properly captured by accelerometer. Self-report measurements also have known limitations that include recall bias, and sitting in transportation was not assessed in this study. Although medication use related to outcomes was introduced into the analyses, we did not have access to information about medical history or family history of cardiovascular and metabolic diseases and therefore could not adjust for them.

## Conclusions

We found that both accelerometer-derived and self-reported sedentary time is associated with cardio-metabolic risk factors in a Japanese working adult population. Subjective and objective measures of sedentary behaviors appear to capture different aspects of behaviors. Sedentary time at the workplace is the main contributor to daily sedentary time in this population. Further efforts to establish a data-processing method integrating objective and subjective measures are needed to assess sedentary time in relation to health outcomes more effectively.

## Electronic supplementary material

Additional file 1: Table S1: Demographic and lifestyle characteristics of the study participants according to accelerometer-derived and self-reported sedentary time; **Table S2.** Cardio-metabolic risk factors profile in the study participants according to accelerometer-derived and self-reported sedentary time. (DOC 154 KB)

## Abbreviations

MVPA: Moderate-to-vigorous physical activity
IPAQ: The International Physical Activity Questionnaire
MET: Metabolic equivalent
JALSPAQ: The Japan Arteriosclerosis Longitudinal Study Physical Activity Questionnaire
BMI: Body mass index
HDL-cholesterol: High-density lipoprotein cholesterol
LDL-cholesterol: Low-density lipoprotein cholesterol
HbA1c: Glycosylated hemoglobin
CES-D: The Center for Epidemiological Studies Depression Scale
SD: Standard deviations
VIF: Variance inflation factor
LPL: Lipoprotein lipase.
