# Supplementary material for: Identifying associations between sedentary time and cardio-metabolic risk factors in working adults using objective and subjective measures: a cross-sectional analysis
Source: BMC Public Health. 2014 Dec 19;14:1307. doi: 10.1186/1471-2458-14-1307 (PMC4302076; doi:10.1186/1471-2458-14-1307)
Supplement: Supplementary file 1 — Additional file 1: Table S1: Demographic and lifestyle characteristics of the study participants according to accelerometer-derived and self-reported sedentary time; Table S2. Cardio-metabolic risk factors profile in the study participants according to accelerometer-derived and self-reported sedentary time. (DOC 154 KB) [file 12889_2014_7436_MOESM1_ESM.doc]

# Additional file 1

## Table S1 - Demographic and lifestyle characteristics of the study participants according to accelerometer-derived and self-reported sedentary time.

|  | **Overall** |  | **Accelerometer-derived** | | | | | |  |  | **Self-reported** | | | | | |  |
| --- | --- | --- | --- | --- | --- | --- | --- | --- | --- | --- | --- | --- | --- | --- | --- | --- | --- |
| **Variables** |  |  | **T1** |  | **T2** |  | **T3** |  | **P for trend** |  | **T1** |  | **T2** |  | **T3** |  | **P for trend** |
|  | **n** | **%** |  |  |  |  |  |  |  |  |  |  |  |  |  |  |  |
| N | 661 |  | 221 |  | 220 |  | 220 |  |  |  | 221 |  | 220 |  | 220 |  |  |
| Men | 515 | 77.9 | 156 | 70.6 | 177 | 80.5 | 182 | 82.7 | 0.002 |  | 163 | 73.8 | 174 | 79.1 | 178 | 80.9 | 0.070 |
| Married | 458 | 69.3 | 134 | 60.6 | 163 | 74.1 | 161 | 73.2 | 0.004 |  | 150 | 67.9 | 164 | 74.6 | 144 | 65.5 | 0.584 |
| Occupation |  |  |  |  |  |  |  |  |  |  |  |  |  |  |  |  |  |
| Professionals | 326 | 49.3 | 98 | 44.3 | 97 | 44.1 | 131 | 59.6 | 0.001 |  | 108 | 48.9 | 98 | 44.5 | 120 | 54.5 | 0.234 |
| Clerks | 90 | 13.6 | 30 | 13.6 | 34 | 15.5 | 26 | 11.8 | 0.592 |  | 26 | 11.8 | 42 | 19.1 | 22 | 10.0 | 0.592 |
| Sales | 115 | 17.4 | 65 | 29.4 | 36 | 16.4 | 14 | 6.4 | <0.001 |  | 57 | 25.8 | 35 | 15.9 | 23 | 10.5 | <0.001 |
| Managers | 108 | 16.3 | 14 | 6.3 | 49 | 22.3 | 45 | 20.5 | <0.001 |  | 18 | 8.1 | 41 | 18.6 | 49 | 22.3 | <0.001 |
| Others | 22 | 3.3 | 14 | 6.3 | 4 | 1.8 | 4 | 1.8 | 0.008 |  | 12 | 5.4 | 4 | 1.8 | 6 | 2.7 | 0.113 |
| Educational attainment (>college/university) | 375 | 56.7 | 95 | 43.0 | 131 | 59.6 | 149 | 67.7 | <0.001 |  | 114 | 51.6 | 135 | 61.4 | 126 | 57.3 | 0.227 |
| Current drinker | 434 | 65.6 | 139 | 62.9 | 152 | 69.1 | 143 | 65.0 | 0.641 |  | 133 | 60.2 | 151 | 68.6 | 150 | 68.2 | 0.077 |
| Current smoker | 188 | 28.4 | 64 | 29.0 | 67 | 30.5 | 57 | 25.9 | 0.478 |  | 64 | 29.0 | 55 | 25.0 | 69 | 31.4 | 0.577 |
| Medication |  |  |  |  |  |  |  |  |  |  |  |  |  |  |  |  |  |
| Anti-hypertensive | 51 | 7.7 | 12 | 5.4 | 26 | 11.8 | 13 | 5.9 | 0.848 |  | 18 | 8.1 | 16 | 7.3 | 17 | 7.7 | 0.869 |
| Anti-diabetic | 13 | 2.0 | 5 | 2.3 | 3 | 1.4 | 5 | 2.3 | 0.995 |  | 5 | 2.3 | 4 | 1.8 | 4 | 1.8 | 0.737 |
| Lipid-lowering | 30 | 4.7 | 8 | 3.6 | 9 | 4.1 | 13 | 5.9 | 0.248 |  | 7 | 3.2 | 15 | 6.8 | 8 | 3.6 | 0.811 |
|  | **Mean** | **SD** |  |  |  |  |  |  | **P for trend** |  |  |  |  |  |  |  | **P for trend** |
| Age, years | 42.4 | 8.6 | 40.4 | 9.8 | 43.3 | 8.4 | 43.6 | 6.9 | <0.001 |  | 41.9 | 9.3 | 43.1 | 8.3 | 42.4 | 8.1 | 0.352 |
| Energy intake, kcal/day | 1829.7 | 581.2 | 1792.8 | 586.8 | 1843.6 | 593.5 | 1852.7 | 563.6 | 0.249 |  | 1859.1 | 572.5 | 1806.1 | 553.4 | 1823.6 | 617.3 | 0.315 |
| Saturated fat, % energy intake | 6.3 | 1.8 | 6.4 | 1.7 | 6.1 | 1.7 | 6.3 | 1.8 | 0.693 |  | 6.4 | 1.7 | 6.3 | 1.8 | 6.1 | 1.9 | 0.047 |
| CES-D, point | 10.0 | 7.2 | 10.6 | 7.5 | 9.5 | 6.8 | 9.9 | 7.2 | 0.298 |  | 10.2 | 7.5 | 9.2 | 6.0 | 10.6 | 7.9 | 0.677 |
| Acceletometer |  |  |  |  |  |  |  |  |  |  |  |  |  |  |  |  |  |
| Sedentary time, hours/day | 8.8 | 2.2 | 6.4 | 1.3 | 8.9 | 0.5 | 11.2 | 1.1 | <0.001 |  | 7.7 | 2.1 | 9.1 | 2.0 | 9.6 | 2.1 | <0.001 |
| MVPA, METs·hours/day | 2.7 | 1.6 | 3.2 | 1.8 | 2.6 | 1.4 | 2.4 | 1.4 | <0.001 |  | 3.0 | 1.7 | 2.7 | 1.5 | 2.5 | 1.4 | <0.001 |
| Self-reported |  |  |  |  |  |  |  |  |  |  |  |  |  |  |  |  |  |
| Total sedentary time, hours/day | 8.4 | 3.4 | 6.9 | 3.3 | 8.6 | 3.3 | 9.7 | 2.9 | <0.001 |  | 5.1 | 1.6 | 8.2 | 0.6 | 11.9 | 2.9 | <0.001 |
| Leisure sedentary time, hours/day | 3.2 | 2.6 | 3.0 | 2.2 | 3.2 | 2.7 | 3.3 | 2.7 | 0.519 |  | 1.9 | 1.2 | 2.4 | 1.2 | 5.2 | 3.2 | <0.001 |
| Occupational sedentary time, hours/day | 5.2 | 2.2 | 3.9 | 2.3 | 5.3 | 1.8 | 6.4 | 1.7 | <0.001 |  | 3.2 | 1.8 | 5.8 | 1.3 | 6.7 | 1.7 | <0.001 |
| MVPA, METs·hours/day | 0.8 | 1.4 | 0.6 | 1.2 | 0.8 | 1.4 | 0.9 | 1.5 | 0.011 |  | 0.8 | 1.2 | 0.8 | 1.3 | 0.8 | 1.6 | 0.569 |

*Abbreviations*: CES-D, the Center for Epidemiological Studies Depression Scale; MVPA, moderate-to-vigorous physical activity; METs, metabolic equivalent.

Cut-off points of sedentary time were 7.99 and 9.76 hours/day for accelerometer, and 7.14 and 9.28 hours/day for self-report, respectively.

## Table S2 - Cardio-metabolic risk factors profile in the study participants according to accelerometer-derived and self-reported sedentary time.

|  | **Overall** |  | **Accelerometer-derived** | | | | | |  |  | **Self-reported** | | | | | |  |
| --- | --- | --- | --- | --- | --- | --- | --- | --- | --- | --- | --- | --- | --- | --- | --- | --- | --- |
| **Variables** |  |  | **T1** |  | **T2** |  | **T3** |  | **P for trend** |  | **T1** |  | **T2** |  | **T3** |  | **P for trend** |
| Body mass index, kg/m^2^ | 22.8 | 3.4 | 22.3 | 3.5 | 23.3 | 3.3 | 23.0 | 3.3 | 0.015 |  | 22.6 | 3.4 | 23.0 | 3.3 | 22.9 | 3.5 | 0.385 |
| Waist circumference, cm | 81.9 | 8.8 | 80.6 | 8.8 | 83.0 | 8.9 | 81.7 | 8.6 | 0.430 |  | 81.4 | 8.9 | 82.3 | 9.2 | 81.8 | 8.4 | 0.787 |
| Systolic blood pressure, mmHg | 120.3 | 14.2 | 119.8 | 13.5 | 121.1 | 14.6 | 120.1 | 14.6 | 0.852 |  | 119.6 | 14.8 | 121.7 | 14.9 | 119.8 | 13.0 | 0.508 |
| Diastolic blood pressure, mmHg | 76.6 | 11.4 | 76.1 | 11.7 | 77.2 | 11.1 | 76.5 | 11.4 | 0.572 |  | 75.5 | 12.0 | 77.5 | 11.5 | 76.7 | 10.7 | 0.177 |
| Triglyceride, mg/dL | 93.2 | (89.5 to 97.1) | 80.7 | (77.3 to 84.2) | 101.3 | (97.3 to 105.5) | 99.2 | (95.7 to 102.9) | <0.001 |  | 87.3 | (83.7 to 91.1) | 94.8 | (91.1 to 98.7) | 97.9 | (94.1 to 101.9) | 0.038 |
| HDL-cholesterol, mg/dL | 62.9 | 16.0 | 66.3 | 16.5 | 62.0 | 16.4 | 60.3 | 14.6 | <0.001 |  | 65.2 | 16.3 | 62.1 | 16.1 | 61.3 | 15.4 | 0.011 |
| Total:HDL ratio | 3.5 | 1.0 | 3.2 | 0.9 | 3.6 | 1.0 | 3.5 | 1.0 | 0.002 |  | 3.4 | 1.0 | 3.5 | 1.0 | 3.6 | 1.0 | 0.045 |
| LDL-cholesterol, mg/dL | 120.9 | 29.5 | 118.9 | 29.4 | 124.0 | 30.1 | 119.8 | 28.7 | 0.850 |  | 121.7 | 28.8 | 119.5 | 30.3 | 121.5 | 29.3 | 0.947 |
| Blood Glucose, mg/dL | 96.2 | (95.2 to 97.2) | 94.2 | (93.2 to 95.2) | 97.4 | (96.3 to 98.5) | 97.1 | (96.1 to 98.1) | <0.001 |  | 95.0 | (93.9 to 96.0) | 96.0 | (95.0 to 96.9) | 97.7 | (96.6 to 98.8) | 0.004 |
| HbA1c, % | 5.3 | (5.3 to 5.4) | 5.2 | (5.1 to 5.2) | 5.4 | (5.4 to 5.5) | 5.5 | (5.5 to 5.6) | <0.001 |  | 5.2 | (5.2 to 5.3) | 5.4 | (5.3 to 5.4) | 5.4 | (5.3 to 5.5) | 0.090 |

*Abbreviations*: HDL-cholesterol, high-density lipoprotein cholesterol; LDL-cholesterol, low-density lipoprotein cholesterol; HbA1c, glycosylated hemoglobin.

Cut-off points of sedentary time were 7.99 and 9.76 hours/day for accelerometer, and 7.14 and 9.28 hours/day for self-report, respectively. N=584 for waist circumference and N=357 for HbA1c due to missing values. Data on triglyceride, blood glucose, and HbA1c were expressed as geometric mean and 95% confidence interval.
